# Interaction of Rio1 Kinase with Toyocamycin Reveals a Conformational Switch That Controls Oligomeric State and Catalytic Activity

**DOI:** 10.1371/journal.pone.0037371

**Published:** 2012-05-22

**Authors:** Irene N. Kiburu, Nicole LaRonde-LeBlanc

**Affiliations:** Department of Chemistry and Biochemistry, Center for Biomolecular Structure and Organization, and the University of Maryland Marlene and Stewart Greenebaum Cancer Center, University of Maryland, College Park, Maryland, United States of America; Nagoya University, Japan

## Abstract

Rio1 kinase is an essential ribosome-processing factor required for proper maturation of 40 S ribosomal subunit. Although its structure is known, several questions regarding its functional remain to be addressed. We report that both *Archaeoglobus fulgidus* and human Rio1 bind more tightly to an adenosine analog, toyocamycin, than to ATP. Toyocamycin has antibiotic, antiviral and cytotoxic properties, and is known to inhibit ribosome biogenesis, specifically the maturation of 40 S. We determined the X-ray crystal structure of toyocamycin bound to Rio1 at 2.0 Å and demonstrated that toyocamycin binds in the ATP binding pocket of the protein. Despite this, measured steady state kinetics were inconsistent with strict competitive inhibition by toyocamycin. In analyzing this interaction, we discovered that Rio1 is capable of accessing multiple distinct oligomeric states and that toyocamycin may inhibit Rio1 by stabilizing a less catalytically active oligomer. We also present evidence of substrate inhibition by high concentrations of ATP for both archaeal and human Rio1. Oligomeric state studies show both proteins access a higher order oligomeric state in the presence of ATP. The study revealed that autophosphorylation by Rio1 reduces oligomer formation and promotes monomerization, resulting in the most active species. Taken together, these results suggest the activity of Rio1 may be modulated by regulating its oligomerization properties in a conserved mechanism, identifies the first ribosome processing target of toyocamycin and presents the first small molecule inhibitor of Rio1 kinase activity.

## Introduction

Protein kinases are involved in a large variety of cellular processes including regulation of gene expression, DNA transcription, signal transduction, cell cycle progression and ribosome biogenesis [1]–[3]. In eukaryotes, protein kinases are among the largest superfamilies with over 500 members identified in the human genome [4]. Of these, 40 are classified as atypical protein kinases (aPKs), while the rest are considered classical eukaryotic protein kinases (ePKs) [4]. Both classes of protein kinases catalyze the phosphorylation of serine, threonine or tyrosine residues.

One family of aPKs is the RIO kinase family that comprises Rio1, Rio2, Rio3 and a group found only in some eubacteria, RioB [5]. Rio1 and Rio2 are highly conserved and present in all archaeal and eukaryotic organisms, whereas Rio3 is only found in metazoa [5]. Previously solved crystal structures of *Archaeoglobus fulgidus* Rio1 (afRio1) and Rio2 (afRio2) have shown the RIO domain is a trimmed version of the ePK catalytic domain with an additional conserved N-terminal α-helix [5], [6]. The ePK catalytic domain is comprised of 250 to 300 amino acids consisting of twelve subdomains that are composed of conserved structural elements [7]. The twelve subdomains include a nucleotide-binding loop, a hinge region between the N- and C-terminal domains that participates in binding the adenine moiety of ATP, a catalytic loop consisting of catalytic Asp and Asn residues, a metal binding loop (DFG loop), substrate binding subdomains and the activation loop (APE loop) that is usually involved in regulation of kinase activity [7]. The RIO domain lacks the activation loop, most of the surface required for peptide substrate binding and recognition in ePK and binds nucleotides in a different conformation compared to the ePKs, suggesting a difference in the substrate interactions and catalytic mechanism [5], [6]. All RIO kinases purified to date are capable of autophosphorylation on serine at sites of varying sequence [5], [8], [9], and recently, it was reported that Rio1 from the archaeon *Haloferax volcanii* is capable of phosphorylating α1, a 20 S proteosome core particle subunit [10]. Rio1 autophosphorylates in trans, such that one molecule of Rio1 will autophosphorylate another [9].

Rio1 kinase is the founding member of the RIO family [8]. Rio1 is involved in ribosome biogenesis, cell cycle progression and chromosome maintenance [8], [11], [12]. Studies have shown that *Saccharomyces cerevisiae* (yeast) Rio1 is an essential gene and a non-ribosomal factor required for the processing of 20 S pre-rRNA to mature 18 S rRNA, the RNA component of the 40 S subunit. Depletion of Rio1 in cells leads to accumulation of 20 S pre-rRNA and cell cycle arrest [13]. Ribosome biogenesis is a highly complex, well-coordinated process in cells involving a large number of processing factors. In eukaryotic cells, it is estimated that over 200 non-ribosomal protein factors are required, including endo- and exonucleases, chaperones, RNA helicases, RNA modifying enzymes and small nucleolar ribonucleoprotein particles (snoRNPs) [3], [14], [15]. Ribosome biogenesis is also tightly linked to cell cycle progression and proliferation [16], [17]. In fact, a distinguishing characteristic of cancer cells is enlarged nucleoli - the compartment where rRNA synthesis and processing occurs [18]. Therefore, modulating the rate of ribosome biogenesis is likely to regulate cell proliferation. One possible avenue for modulating ribosome biogenesis is through targeting and inhibiting proteins required for rRNA processing such as Rio1 kinase.

**Figure 1:**
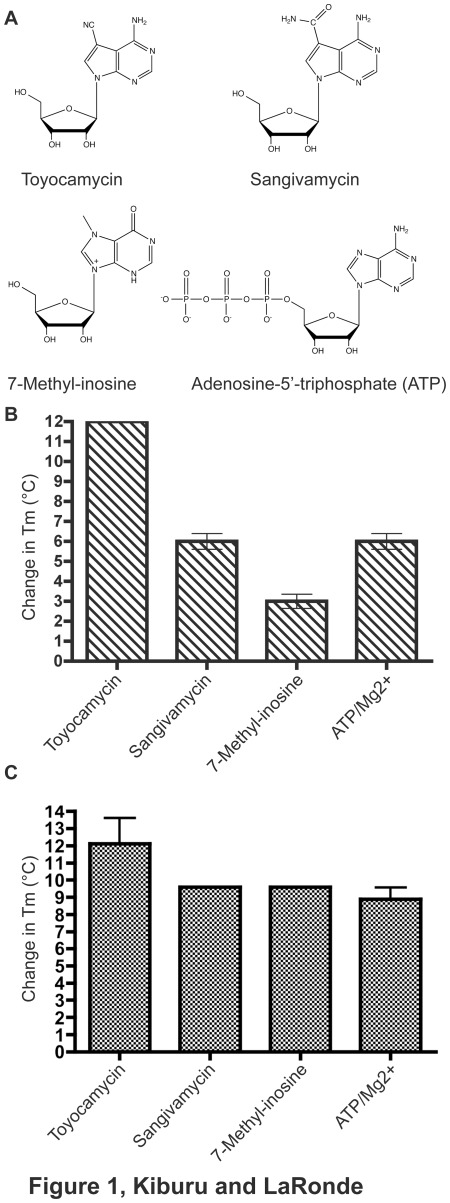
Adenosine analogs screened for Rio1 binding activity. A. Chemical structures of molecules used in this study. Thermal shift data for B. afRio1 and C. hRio1. Difference between melting temperatures (T_m_) for unbound afRio1 and complexes with each compound plotted. Actual thermal shift plots provided in Fig. S1.

In initial attempts to find starting fragments targeting the afRio1 ATP-binding site, we employed a structure-based approach to identify adenosine analogs that we would predict to bind tightly to Rio1. We identified toyocamycin (Fig. 1A), a 7-cyano-7-deazaadenosine produced by *Streptomyces toyocaensis* (a gram-positive bacterium) as a tight binding adenosine analog. Previous studies that date back to 1968 showed that low concentrations of toyocamycin selectively inhibited 28 S and 18 S rRNA maturation in mammalian cells [19]. Further investigations on the effect of toyocamycin on several cancer cell lines such as HeLa cells, colon carcinoma cells and chick embryo cells infected with MC29 Avian Leukosis virus also revealed the inhibition of rRNA processing [20]–[22]. Toyocamycin is also capable of inhibiting kinases such as phosphatidylinositol kinase, rhodopsin kinase and adenosine kinase from Mycobacterium tuberculosis [23]–[25].

We hereby present a crystal structure of toyocamycin bound to afRio1 at 2.0 Å resolution and employ structural analysis to characterize the interactions. This complex structure shows binding of toyocamycin to the ATP binding pocket and further characterization on the effect of toyocamycin on afRio1 enzymatic activity reveals inhibition by association of oligomers. Interaction of afRio1 with toyocamycin and presence of multiple oligomeric states appears to be conserved in human Rio1 kinase (hRio1). We therefore identify a tight binding analog and present the first report of a compound that inhibits the phosphorylation activity of Rio1 kinase. We also directly link toyocamycin to rRNA processing inhibition via inhibition of Rio1, a ribosome-processing factor, and our results suggest a role for Rio1 autophosphorylation in modulation of the kinase’s oligomeric properties.

## Materials and Methods

### Protein Expression and Purification of afRio1 Kinase and Y200D mutant

The full-length afRio1 was expressed and purified as previously described [9]. Site- directed mutagenesis was performed using the QuikChange™ Lightning kit (Agilent Technologies) according to the manufacturer’s instructions. Mutagenic primers used were 5′GGTGCATGCGGATCTGAGCGAAGATAACATTATGTATATTGATAAA 3′as the sense primer and 5′ TTTTATCAATATACATAATGTTATCTTCGCTCAGATCCGCATGCACC 3′ as the antisense primer. Protein expression and purification of afRio1Y200D followed the same protocol as the full-length afRio1 [9] with one modification. After lysis, the cells were heated at 68°C instead of 75°C for 15 minutes to denature *E.* *coli* proteins. AfRio1 purified under these conditions is not autophosphorylated, as confirmed by mass spectrometry (data not shown) and structural data previously reported [26].

### Protein Expression and Purification of hRio1

A construct of hRio1 containing residues 106 to 494 was expressed and purified as previously reported for afRio1 [9] with a few modifications. After lysis, the cells were immediately centrifuged at 18,000 rpm for 30 minutes at 4°C with no denaturation step. The buffer used during the size exclusion purification step, contained 50 mM Tris pH 8.0, 200 mM NaCl, and 0.2% β-mercaptoethanol. To prevent autophosphorylation, the protein is purified in the presence of 1 mM adenosine, which prevents autophosphorylation.

### Thermofluor Assays

All afRio1 assays were carried out in 50 mM Tris pH 8.0, 75 mM NaCl and 2 mM MgCl_2_. Reactions of 50 µl of buffer containing afRio1 and adenosine analogs at a concentration of 1.0 mg/ml protein and 2 mM ligand respectively were incubated at 25°C for 30 minutes. 50 µl of Sypro-Orange™ dye (Sigma-Aldrich) diluted to 2X in reaction buffer (above) was then added to the reactions. As a reference, reactions were also set up in which no ligand was added. The reactions were heated from 30°C to 98°C using a Bio-Rad Mini-Opticon™ thermal cycler at a rate of 0.2°C per second with an initial holding time of 2 minutes. HRio1 assays followed the same protocol as afRio1 and were carried out in a buffer containing 50 mM Tris pH 8.0, 200 mM NaCl and 0.2% β-mercaptoethanol. HRio1 reactions were initially incubated at 4°C for 30 minutes and then heated from 4°C to 98°C using the thermal cycler. The change in fluorescence intensity (I) was monitored as the temperature increased. All measurements were done in triplicates and the melting temperature (T_m_) was determined as the inflection point of the melting curve (or the peak of dI/dT). Shifts in melting temperature (T_m_) were calculated as the difference between the T_m_ of the control reaction (unbound afRio1/hRio1) and T_m_ of the reaction containing the compound of interest.

### Co-crystallization of afRio1 with Toyocamycin

Purified afRio1 was concentrated to 21 mg/ml in a buffer containing 10 mM Tris pH 8.0, 10 mM NaCl, and 0.2% β-mercaptoethanol. AfRio1 was incubated with 2 mM toyocamycin at 25°C for one hour and the complex was subjected to robotic sparse matrix screening in 96 well plates using the sitting drop method in three different afRio1-toyocamycin complex to well solution ratios (1∶2, 1∶1, 2∶1) using the Phoenix™ Liquid Handing system (Art Robbins). We used six commercially available screening matrices; Index™ (Hampton Research), Cryos Suite™ (Qiagen), PEGs Suite™ (Qiagen), Wizard™ I, II and III (Emerald Biosystems). The plates were incubated at 20°C and crystals were observed after seven days in the PEGs Suite™ screen. These crystals grew in the 1∶2 protein to well solution ratio in 0.2 M Mg (CH_3_COO)_2_, 24% PEG 3350, pH 8.0. The crystals were optimized using the hanging drop method with a precipitant range of 14%–32% PEG 3350 and the salt range was varied from 0.15 M to 0.3 M. The optimized trays were incubated at 20°C and we were able to obtain good quality crystals (120×50×5 µm) after one week.

### Crystal Structure Determination and Refinement

AfRio1-toyocamycin crystals were flash frozen in well solution containing 20% (v/v) glycerol. Data was collected at 100 K at the NE-CAT beamline at the Advanced Photon Source (APS), Argonne, IL, USA. All data was indexed, integrated and scaled using HKL2000 program [27]. Data statistics are provided in Table 1. A previously solved structure of afRio1 bound to ATP and Mn^2+^ (PDB ID: 1ZP9) was used as a molecular replacement model to phase the afRio1-toyocamycin complex structure. The afRio1-toyocamycin data set was phased using MOLREP [28]–[30] as part of CCP4i and subjected to several rounds of building using the model-building program COOT [31] and several rounds of refinement using REFMAC5 [32] and PHENIX [33], [34]. Data collection and refinement statistics are provided in Table 1. The coordinates and structure factors have been deposited in the Protein Data Bank (PDB ID: 3RE4).

**Table 1:** Data Collection and Refinement Statistics.

Data collection:	afRio1/Toyocamycin
Space group	P2_1_
Cell dimensions	
*a*, *b*, *c* (Å)	52.8, 72.6, 60.6
β (°)	90.2
Molecules/Asym. unit	2
Wavelength (Å)	0.97919
Resolution (Å)	40-2.00
*R*_sym_ (last shell)	0.069 (0.282)
*I*/σ*I*	18.2 (5.5)
Completeness (%)	97.7 (96.1)
Redundancy	4.0 (3.9)
**Refinement:**	
Resolution (Å)	34.9-2.00
Residues	503
*R*_work_/*R*_free_ (%)	21.5/26.0
Waters	175
*Mean B*-factors (Å)^2^ Residues, Water, Ligands	30.6, 34.6, 27.6
RMS deviations:	
Bond lengths (Å)	0.011
Bond angles (°)	1.385
Ramachandran plot:	
Favored	95.1%
Additional Allowed	4.9%
Disallowed	0.0%

### Steady State Enzymatic Assays

In order to determine the K_m_ of afRio1 for ATP, 0.5 µg of purified afRio1 and 10 µg of MBP was added to a 30 µl reaction volume containing 50 mM Tris pH 8.0, 50 mM NaCl, 2 mM MgCl_2_ and 0.2% β-mercaptoethanol. The final concentration of ATP added was varied from 1×10^−6 ^M to 7.9×10^−9 ^M. For each reaction, 5 µCi of γ-^32^P labeled ATP was added and the specific activity determined for each concentration. Each reaction was incubated at 37°C and stopped at specific time points (0, 1, 2, 4, 6, 8, 10, 15, 20, 30 and 40 mins) by adding 8 µl of SDS-PAGE loading dye and heating the reaction at 95°C for 5 minutes. All reactions were carried out in triplicates. The reaction mixtures were run on NuPAGE™ 4–12% Bis-Tris denaturing gels (Invitrogen) at 200 V for 30 mins in order to separate afRio1, ATP and MBP. The gels were dried and exposed on a phosphorimager screen for 15 hours. Total intensity was quantified using ImageQuant to determine moles of γ-^32^P incorporated into MBP. For the inhibition studies, the same protocol was followed with the addition of toyocamycin to the reaction assay. Inhibition studies were carried out at three different concentrations of toyocamycin 20 nM, 40 nM and 60 nM. For autophosphorylated afRio1 samples, the protein was first incubated at 37°C in 50 mM Tris pH 8.0, 50 mM NaCl, 2 mM MgCl_2_ and 0.2% β-mercaptoethanol with 1 µM ATP for 1 hr and further purified by size exclusion chromatography using the reaction buffer in the absence of ATP. The enzymatic assays followed the same protocol as the unphosphorylated afRio1.

### Substrate Inhibition by High Concentrations of ATP

**Figure 2:**
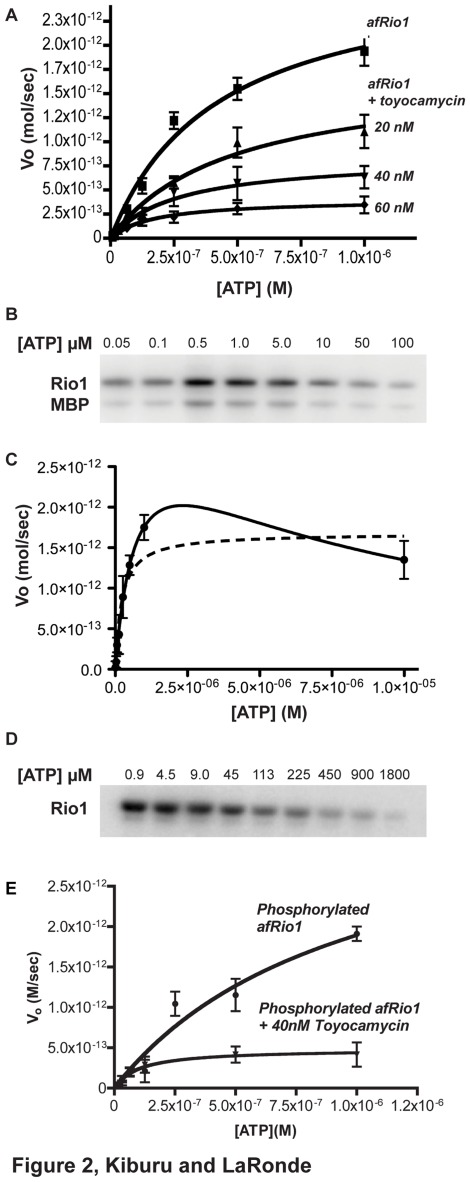
Steady state analyses of afRio1. A. V_o_ vs [ATP] curves for afRio1 in the presence and absence of 20, 40 and 60 nM toyocamycin. B. Autoradiogram showing the transfer of ^32^P-labelled phosphate to afRio1 (by autophosphorylation) and MBP as a function of increasing ATP concentration [ATP]. C. V_o_ vs [ATP] curves for afRio1 showing substrate inhibition at high concentrations of ATP. D. Autoradiogram showing autophosphorylation of human Rio1 as a function of increasing [ATP]. E.V_o_ vs [ATP] curves for phosphorylated afRio1 in the presence and absence of 40 and 60 toyocamycin.

Data shown in Fig. 2B and C were produced by assays that followed the same protocol as steady state enzymatic assays with a few modifications. 1 µg of afRio1 and 5 µg of MBP were used in the reaction and ATP concentrations were varied from 1×10^−4^ M to 1.0×10^−7^ M. All reactions were incubated at 37°C for 45 minutes. For the hRio1 assay, 5 µg of hRio1 was added to a buffer containing 20 mM Tris pH 8.0, 100 mM NaCl, and 2 mM MgCl_2_ and different amounts of ATP concentrations that varied from 1.8×10^−3^ M to 9.0×10^−7^ M. The hRio1 reactions were incubated at 25°C for 1 hour.

### Sedimentation Equilibrium Experiments

All afRio1 samples were dialyzed against 50 mM Tris pH 8.0, 50 mM NaCl, 2 mM MgCl_2_ and 0.2% β-mercaptoethanol. The samples were prepared in three different concentrations of 0.5, 0.25 and 0.125 µg/µl each. In order to ensure that there was no absorbance interference from toyocamycin, ATP and ADP, an absorbance spectrum at different concentrations for each compound was collected at 280 nm. The highest concentration that showed no absorbance at 280 nm was 1×10^−5^ M for all three compounds, which is at saturation levels for afRio1 binding. This concentration was used in the experiments. For autophosphorylated afRio1 samples, the protein was first incubated at 37°C in sedimentation buffer with 1 µM ATP for 1 hr and further purified by size exclusion chromatography in the sedimentation equilibrium buffer. Analysis of autophosphorylated afRio1 in the presence of ATP or toyocamycin, the appropriate compound was added to the sample for a final concentration of 1×10^−5^ M. As with unphosphorylated afRio1 the protein samples were analyzed at three different concentrations of 0.5, 0.25 and 0.125 µg/µl each. The experiments were carried out at 20°C in a Beckman XL-I Analytical Ultracentrifuge and data collected at 280 nm using six channel Epon centerpieces with a 12 mm path-length containing 110 µl samples and 120 µl buffer references and run at three speeds 18000 rpm, 22000 rpm and 26000 rpm. Data was analyzed using the software, WinNonlin [35].

### Size Exclusion Chromatography of afRio1 and hRio1

In order to determine the oligomeric states of ligand free, adenosine-bound, ATP-bound and autophosphorylated Rio1, the samples with a final protein concentration of 3 µg/µl were analyzed using size exclusion chromatography. Autophosphorylated afRio1 was prepared as described in the sedimentation equilibrium section (above). For autophosphorylated hRio1 sample, the protein was incubated at 25°C for 1 hr in 50 mM Tris pH 8.0, 200 mM NaCl, 2 mM MgCl_2_ and 0.2% β-mercaptoethanol. An analytical Superdex™ 200 column (GE Healthcare) was equilibrated with a buffer containing 50 mM Tris pH 8.0, 200 mM NaCl and 0.2% β-mercaptoethanol. The calibration proteins, ribonuclease, ovalbumin, conalbumin and aldolase (GE Healthcare), were diluted with 50 mM Tris pH 8.0 and 200 mM NaCl for a final concentration of 3 µg/µl. All four calibration proteins were mixed together, centrifuged to remove any precipitants and loaded onto the column. Finally, the Rio1 protein samples were diluted in equilibration buffer containing 2 mM ligand and loaded onto the column. All size exclusion runs were monitored by absorbance at 280 nm.

## Results

### Identification of Small Molecule Stabilizers of afRio1 Kinase

In an effort to identify molecules that would act as tight binding fragments for the design of specific RIO kinase inhibitors, we tested N7-substituted analogs of adenosine for binding to afRio1. Based on the observation of an open pocket near the N7 of the adenine ring in the crystal structure of the Rio1-ATP binding site, we determined that these analogs would be a good starting point. Using thermal shift assays that monitor the shift in melting temperature (T_m_) of bound afRio1 in comparison to unbound afRio1, we identified two adenosine analogs, sangivamycin and toyocamycin, that resulted in a significant increase in the T_m_ of afRio1 compared to the unbound protein (Fig. 1B, Fig. S1A). The T_m_ of unbound afRio1 is 76°C ±0.4°C and in the presence of ATP and magnesium (Mg^2+^) the T_m_ shifts to 82°C ±1.0°C, a 6°C increase. The T_m_ in the presence of sangivamycin and toyocamycin was 82°C ±1.2°C and 88°C ±0.1°C respectively, which is similar to ATP/Mg^2+^ in the case of sangivamycin, and a 12°C shift for toyocamycin (Fig. 1B). We also tested 7-methylinosine, which we did not expect to bind optimally because of the carbonyl moiety at the N6 position. We observed a 3°C shift, indicating weaker binding than ATP/Mg^2+^. Previous afRio1 crystal structures that have been solved in the presence of adenosine, ADP and ATP show that N6 position of the adenine moiety requires a hydrogen donor to interact with the peptidyl carbonyl oxygen of Glu 148 [26]. The data was a clear indication that toyocamycin binds to afRio1 more tightly than ATP/Mg^2+^, despite the lack of a triphosphate moiety and hence absence of metal ion interactions. The same compounds used in afRio1 assay (Fig. 1A) were tested for binding to hRio1 kinase. Unbound hRio1 kinase had a T_m_ of 41.1±1.1°C. The T_m_ increased to 53.2±1.8°C in the presence of toyocamycin, a 12.1°C increase, similar to what is observed with afRio1. In the presence of either sangivamycin or 7-methylinosine, the T_m_ was 50.7°C, showing an increase of 9.6°C. When ATP/Mg^2+^ is added to hRio1, the T_m_ is 50.0±1.2°C corresponding in an 8.9°C shift. Taken together this data shows conserved high affinity for toyocamycin in both the archaeal and human Rio1.

### Structure Determination of the AfRio1/Toyocamycin Complex

**Figure 3:**
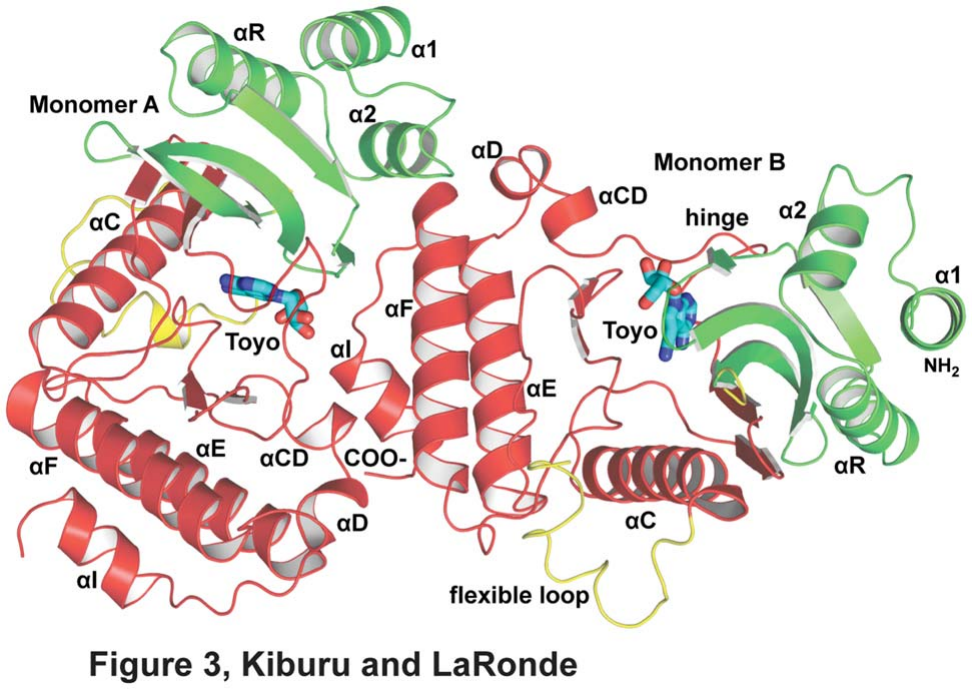
Dimer of afRio1/toyocamycin (toyo) complex observed in the asymmetric unit.

In order to study how toyocamycin is able to stabilize afRio1 to a greater extent than ATP/Mg^2+^, we determined the crystal structure of afRio1 bound to toyocamycin. The completely refined structure contained two molecules of the afRio1/toyocamycin complex per asymmetric unit with residues 3-258 for each molecule (Fig. 3). The afRio1 structure consists of an N-terminal domain (N-lobe) that consists of a α-helical region and a twisted β-sheet that contains the phosphate interacting loop (P-loop) and an α-helical C-terminal region (C-lobe) that houses the catalytic and metal binding residues. The N- and C-lobes are connected by a hinge region, and between the two domains is a cleft that buries the 7-cyano-7-deazaadenine ring of toyocamycin. Rio1 has a flexible loop consisting of Arg 83-Glu 111 that changes conformation when ATP/Mn^2+^ is bound. In the afRio1/toyocamycin complex, part of that flexible loop, residues 86–90 in chain A and residues 86–93 in chain B, are not observed in the electron density map. This is similar to the structure of ATP/Mn^2+^ bound to afRio1 for which no electron density was observed for residues 85–91. The RMSD between the two molecules in the asymmetric unit is 0.024 Å^2^ for 240 residues (Fig. S2).

### Interactions Between Toyocamycin and Rio1

**Figure 4:**
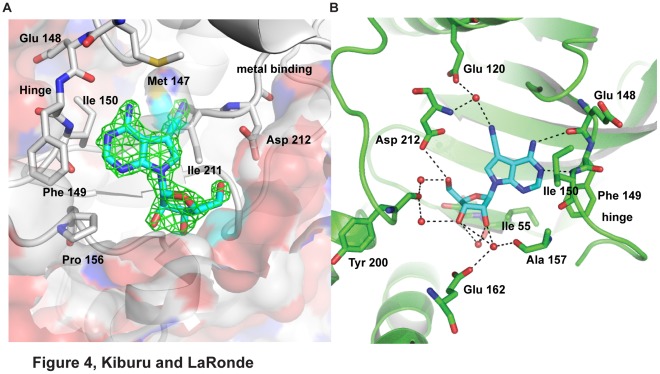
Interactions between afRio1 and toyocamycin. A. Active site of afRio1 overlaid with difference density (F_o_-F_c_) map calculated using model in which toyocamycin (toyo) is omitted (contoured at 2.5σ). Some of the residues lining the hydrophobic pocket of the active site are labeled. B. Hydrogen bonding interactions (dashed lines) observed between toyocamycin and afRio1.

A difference electron density (Fo-Fc) map, generated by refining the search model in the absence of any ligand revealed an electron density for toyocamycin (Fig. 4A). Each toyocamycin molecule in the asymmetric unit had a low average B-factor of 27.5 and 27.6 for monomer A and B respectively. An overlay of the toyocamycin and ATP binding sites demonstrates that toyocamycin binds to the same site as ATP (Fig. S3). Toyocamycin is buried in the active site pocket that features hydrophobic contact with Val 63, Ile 55 and Ala 78 from the N-lobe, Pro 156, Met 147,Phe 149 and Ile 150 from the hinge, and Met 203 and Ile 211 from the C-lobe. In addition, toyocamycin participates in ten hydrogen bond interactions. There is one direct interaction from the peptidyl carbonyl oxygen of Glu 148 to the amino group N6 and another from the indole nitrogen, N1 to the peptidyl amine group of Ile 150 (Fig. 4B). This is similar to the binding mode observed in this region with ATP [5]. Another hydrogen bond is seen between the 7-cyano group to a water molecule contacting the carbonyl side chain of Glu 120 and peptidyl amine group of Asp 212 (Fig. 4B). This water molecule is seen in all available structures of afRio1 and in the ATP/Mn^2+^ bound afRio1 it mediates a hydrogen bond between Glu 120 and γ-phosphate. The ribose 3′ hydroxyl participates in water-mediated contacts with the side chain of Glu 162, with the peptidyl carbonyl oxygen of Tyr 200 and with the peptidyl carbonyl oxygen of Ile 55. The ribose 2′ hydroxyl participates in water-mediated hydrogen bonds with the peptidyl carbonyl oxygens of Ile 55, Ala 157 and Glu 162. These water-mediated interactions are not observed in the ATP/Mn^2+^ bound to afRio1 structure. The 5′ hydroxyl is in hydrogen bonding distance of Asp 212 and involved in a water-mediated hydrogen bond interaction with the peptidyl carbonyl oxygen of Tyr 200 (Fig. 4B). In the ATP/Mn^2+^ bound to afRio1 complex, Asp 212 side chain directly hydrogen bonds with the γ-phosphate, and is liganded to the Mn^2+^ that coordinates the α- and β-phosphates.

### Steady state kinetic analyses of afRio1

In order to determine whether toyocamycin acts as a strict competitive inhibitor of afRio1 kinase activity, we determined the steady state kinetic parameters for afRio1 in the presence and absence of toyocamycin. We determined the K_m_ for ATP and V_max_ of afRio1 in an assay that measured the incorporation of γ-^32^P from ATP into myelin basic protein (MBP), a commonly used general kinase substrate that is measurably phosphorylated by afRio1 [26]. The K_m,ATP_ was 0.4 µM (±0.1) whereas the V_max_ was 2.6 pmol/sec (Table 2, Fig. 2A), determined by non-linear least squares fits of the Michaelis-Menten equation V_o_ = (V_max_[S])/(K_m_ + [S]) to plots of the initial velocity (V_o_) as a function of ATP concentration ([ATP] or [S]). Standard error estimations are reported from the fit to the mean of triplicate measurements. We also observed that Rio1 kinase catalytic activity begins to decrease at high [ATP] (Fig. 2B). At [ATP] above 0.1 µM, kinase activity begins to drop until at 100µM it becomes almost unmeasurable. Figure 2C shows a curve fit using a substrate inhibition model with the equation V_o_ =  (V_max_[S])/(K_m_ + [S]([1+[S]/K_i,ATP_)) was used to fit the V_o_ vs [S] plots, where [S] is [ATP], and K_i,ATP_ is the substrate inhibition constant. The K_m_,_ATP_ determined using this model was 0.8 µM (±0.2), and K_i,ATP_ was 7.1 µM. This substrate inhibition behavior is also observed in similar assay using the human Rio1 kinase (Fig. 2D). Therefore, our analyses of steady state parameters were limited to data collected at low [ATP].

**Table 2:** Steady State Analysis for afRio1.

	afRio1	+20 nM Toyocamycin	+40 nM Toyocamycin	+60 nM Toyocamycin
Km (µM)	0.4 (±0.1)	0.5 (±0.2)	0.2 (±0.03)	0.2 (±0.1)
Vmax(pmol/sec)	2.6 (±0.3)	1.8 (±0.3)	0.6 (±0.03)	0.4 (±0.1)
Ki, (nM)		10.7	26.7	3.8
Ki’(nM)		40	11.4	5.5

The steady state kinetic parameters were also determined in the presence of three different concentrations of toyocamycin based on the equilibrium dissociation constant (K_d_) for toyocamycin binding which was estimated to be 40 nM (data not shown). An initial non-linear least squares fit using the Michaelis-Menten equation (V_o_ = (V_max_[S])/(K_m_ + [S]) was performed on the data. At 40 nM, the apparent K_m_ was determined to be 0.2 µM (±0.03) with a V_max_ of 0.6pmol/sec. This shows a 1.8 fold decrease in the K_m_ and a 4.5 fold decrease in the V_max_. A change in both K_m_ and V_max_ is inconsistent with strict competitive inhibition, in which case one would expect an increase in the K_m_ and no change in the V_max_. At 20 nM, the apparent K_m_ is within error of the Km determined without inhibitor and at 60 nM it is lower than at 40 nM (Table 2). The Vmax shows a steady decrease as inhibitor concentration is increased (Table 2 and Fig. 2A). Since toyocamycin decreases Vmax, we tested different inhibition models for a global fit of Vo vs [S] plots for the data sets (excluding [S] above 1 µM). The best global fit was to a mixed inhibition model with the modified Michaelis-Menten equation, Vo = (Vmax [S])/(αKm + α’[S]), where α = 1+([I]/Ki), α’ = 1+([I]/Ki’) and Ki and Ki’ are the inhibition constants. The inhibition constants in the presence of a) 20 nM toyocamycin were 10.7 nM and 40 nM, b) in the presence of 40 nM toyocamycin were 26.7 nM and 11.4 nM, and c) in the presence of 60 nM toyocamycin were 3.8 nM and 5.5 nM as reported on Table 2 (curve-fits shown in Fig. 2A). Steady state kinetics data in the presence of toyocamycin had the best global fit to a mixed inhibition model that encompasses both competitive and uncompetitive inhibition. Crystallographic studies support competitive inhibition as we observe binding of toyocamycin to the ATP pocket, and not to any other site in the presence of a saturating amount of toyocamycin (2 mM), that is above the Kd (40 nM) during the co-crystallization studies. We therefore hypothesized that the apparent mixed inhibition may result from modulation of active sites through oligmerization.

### Determination of the oligomeric states of Rio1

**Figure 5:**
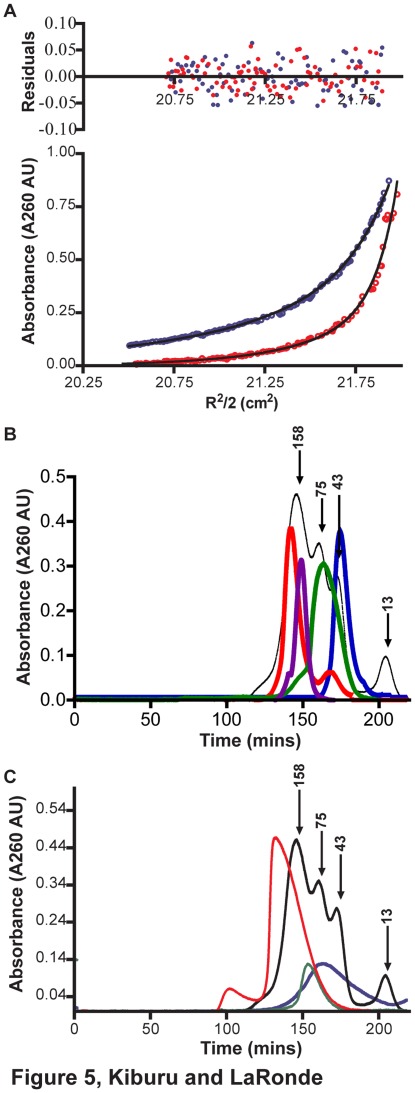
Characterization of the oligomeric states of Rio1. A. Sedimentation equilibrium data collected at 18,000 rpm with residuals and best fit curves for phosphorylated afRio1 (blue dots; single species model) and the afRio1/toyocamycin complex (red dots; monomer-tetramer model) B. Size exclusion chromatography plots for afRio1 in an unphosphorylated state (red), unphosphorylated afRio1 with ATP (purple), phosphorylated afRio1 (blue) and phosphorylated afRio1 with ATP bound (green). C. Size exclusion chromatography plots for hRio1 unphosphorylated state with adenosine bound (red), unphosphorylated hRio1 with ATP bound (green), phosphorylated hRio1 (purple). Plot for standards used in column calibration is overlaid in black with numbers indicating the known molecular weight (kDa) for the standards in each peak.

The steady state kinetic data suggested in addition to competitive inhibition there is inhibition by modulation of separate binding sites in an oligomer. This would imply that toyocamycin binding stabilizes an oligomeric form that is less catalytically active than the free form, the monomer. This allosteric modulation model would also be consistent with the observed ATP-dependent inhibition, if high concentrations of ATP promote oligomerization and stabilize a less active oligomer as well. In order to test this model, we determined the oligomeric states of afRio1 under several conditions. Using analytical ultracentrifugation, we analyzed the oligomeric states of both the autophosphorylated and unphosphorylated forms of afRio1 in the presence and absence of ATP/Mg^2+^ and toyocamycin by sedimentation equilibrium analysis. The average molecular weights, determined by fitting a single species (monomer) model to the absorbance data collected at three different speeds (18000, 22000 and 26000 rpm), are shown in Table 3. In almost every case except for the autophosphorylated unliganded afRio1, a single species model resulted in a poor fit as determined by evaluation of both the residuals and (variance)^ 1/2^. Therefore, the data were fit to various oligomeric state models (monomer, monomer-dimer, monomer-dimer-trimer, monomer-dimer-tetramer, and monomer-tetramer equilibria). The smallest and biggest species are observed with autophosphorylated afRio1 and, the unphosphorylated toyocamycin bound afRio1, respectively (Fig. 5A). The rest of the fitted data is shown in supplementary figure S4. The results of the best fits, characterized by the simplest model with the lowest (variance)^ 1/2^ values, are summarized in Table 3. Table S1 provides the (variance)^1/2^ for all oligomerization models tested and supplementary figure S5A-Q shows the residuals. The calculated monomeric molecular weight of afRio1 is 30.1 kDa and the unliganded afRio1 fit to an average molecular weight consistent with a dimer (60.2±1.1 kDa) in the unphosphorylated form, and a monomer (34.1±1.0 kDa) in the phosphorylated form. In the presence of ATP or ADP and Mg^2+^, the average molecular weight was between monomer and dimer (53.6±1.1 kDa) for ATP and (50.8±1.1 kDa) for ADP for the unphosphorylated protein. With toyocamycin present, the average molecular weight was larger than a dimer (71.8±1.2 kDa), and the best fit was obtained using a model that assumed a mixture of monomer and tetramer. In all cases, we cannot definitively rule out the existence of some fraction of dimer, trimer or tetramer, since these are all theoretically possible in this open oligomerization (i.e. an interaction interface is always available in the oligomer). These data shows that unphosphorylated afRio1 forms higher order oligomers and autophosphorylation promotes monomerization. Addition of ATP and toyocamycin to the phosphorylated form fit to a monomer-trimer model and resulted in an increase in the average molecular weights compared to the unligand autophosphorylated protein, the most monomeric form. Taken together these results suggest that both ATP and toyocamycin promote at least dimerization and perhaps even tetramerization of afRio1 consistent with the suggested model for inhibition by both compounds. Size exclusion chromatography data also shows multiple oligomeric states for afRio1 depending on ligand and phosphorylation state. Shown in Figure 5B is an overlay of the chromatograms obtained for the phosphorylated and unphosphorylated protein in the presence and absence of ATP. The ligand-free phosphorylated afRio1 peak runs closest to monomer and is shifted closer to tetramer or trimer when ATP is present. The unphosphorylated afRio1 shows a species running slightly larger than tetramer and a small amount of dimer. When ATP is added, a broad peak with a mean size of dimer is observed. These data clearly show sampling of a number of different oligomeric states by afRio1 again consistent with the proposed inhibition by modulation of different oligomeric states as described above.

**Table 3:** Analysis of Sedimentation Equilibrium Data for Various afRio1 Complexes.

Sample	Weight Averaged MW (kDa)	Best Fit Model	(Variance) ½	Estimate K_d_ (M)
afRio1	60.2±1.1	Monomer- tetramer	8.0×10^−3^	6.1×10^−6^
afRio1+ toyocamycin	71.8±1.2	Monomer- tetramer	1.8×10^−2^	1.5×10^−6^
afRio1+ATP	53.6±1.1	Monomer-dimer	2.8×10^−2^	3.1×10^−6^
afRio1+ADP	50.8±1.1	Monomer-dimer	2.4×10^−2^	6.3×10^−6^
Phosphorylated afRio1	34.1±1.0	Monomer	3.1×10^−2^	N/A
Phosphorylated afRio1+ATP	47.6±1.1	Monomer-trimer	8.8×10^−3^	7.0×10^−6^
Phosphorylated afRio1+ toyocamycin	46.2±1.2	Monomer-trimer	1.3×10^−2^	6.4×10^−6^
afRio1 Y200D	70.6±1.1	Monomer- tetramer	9.2×10^−3^	1.0×10^−7^
afRio1 Y200D +toyocamycin	48.2±1.1	Monomer-trimer	9.3×10^−3^	1.2×10^−5^
afRio1Y200D +ATP	46.8±1.1	Monomer-trimer	9.4×10^−3^	7.3×10^−6^

Since ATP also inhibits afRio1 at concentrations above 1 µM (Fig. 2C), we were interested in whether or not a similar mechanism of inhibition is possible for the hRio1. The oligomeric state of hRio1 was analyzed by size exclusion chromatography (Fig. 5C). We were unable to stabilize hRio1 protein sufficiently to allow analysis by analytical ultracentrifugation. Unphosphorylated hRio1 (monomer MW  = 45.1 kDa) in the presence of adenosine eluted as a tetramer (179.1 kDa), and as a mixture of monomer and dimer when phosphorylated (Fig. 5C). Addition of ATP results in a shift in the monomer-dimer equilibrium to mostly dimer (104.3 kDa). This shows sampling of different oligomeric states by hRio1 agreeing with what is observed with afRio1 suggesting that both Rio1 kinases may employ a similar mechanism in the ATP-dependent inhibition.

### Steady state kinetics analysis of autophosphorylated afRio1

Autophosphorylated afRio1 is monomeric in its unliganded form as evident from sedimentation equilibrium experiments. In an effort to determine the steady state parameters of phosphorylated afRio1, the protein was subjected to the same steady state kinetic assay and analysis as the unphosphorylated afRio1. The phosphorylated afRio1 had a K_m_ of 1.0 µM (±0.6) and a V_max_ of 3.8 pmol/sec (Table 4, Fig. 2E). This data suggests that phosphorylated monomeric afRio1, which has a maximum reaction velocity (V_max_) of 3.8 pmol/sec, is more active than the unphosphorylated afRio1, which has a V_max_ of 2.6 pmol/sec. This is consistent with the hypothesis that phophorylation promotes monomerization resulting in a more active form of afRio1 and association of oligomers results in a less active form as is evident with the unphosphorylated afRio1. Inhibition studies were carried out in the presence of 40 nM toyocamycin and the K_m_ and V_max_ decreased with values of 0.1 µM (±0.04) and 0.5 pmol/sec respectively. Therefore toyocamycin also inhibits the phosphorylated form in a similar mechanism as the unphosphorylated afRio1, probably by promoting self-association since toyocamycin promotes oligomerization of phosphorylated afRio1 (Table 3). In addition, a decreased K_m_ value indicates that toyocamycin increases the affinity for ATP, which would be consistent with the promotion of the ATP bound oligomeric form of afRio1.

**Table 4:** Steady State Analysis of Phosphorylated afRio1.

	Phosphorylated afRio1	+40 nM Toyocamycin
K_m_ (µM)	1.0 (±0.6)	0.1 (±0.03)
V_max_ (pmol/sec)	3.8 (±0.1)	0.5 (±0.04)

### Analysis of Interaction Interfaces in Crystal Structures

Having established that afRio1 forms higher order oligomers depending on phosphorylation state and bound ligand, we analyzed crystal structure lattice packing interactions for possible interaction surfaces. Previously determined crystal structures of unphosphorylated afRio1 bound to adenosine (ADE), ATP/Mn^2+^ and ADP/Mn^2+^, and the toyocamycin complex structure were used to compare packing interfaces and identify structural differences that may promote oligomer formation [26]. It was previously reported that the unphosphorylated afRio1 with adenosine bound differed from the ATP/Mn^2+^ bound form in the positioning of the flexible linker. In addition, conformational changes were observed in the N-terminal helices of the N-lobe [9]. In this paper, we report an analysis of interaction interfaces observed in the above structures.

**Figure 6:**
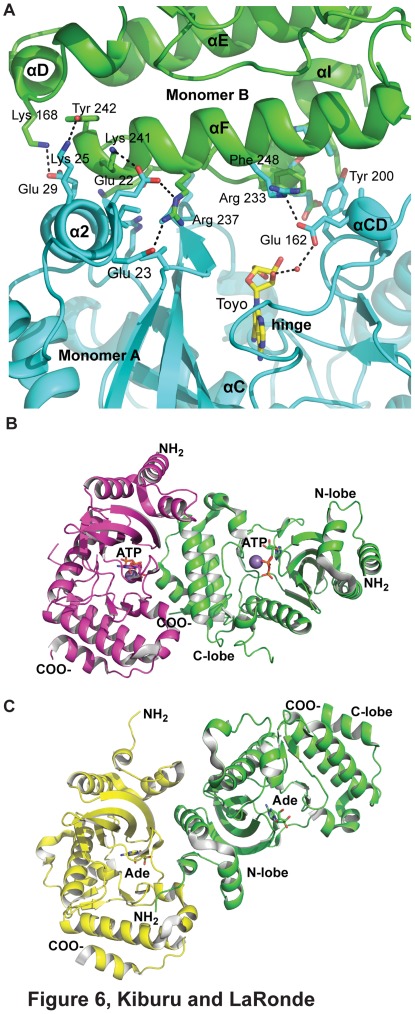
Dimer interfaces of afRio1 complexes. A. Interactions at the afRio1/toyocamycin (Toyo) dimer interface. Separate monomers are colored in cyan and green. Residues shown in stick participate in direct interactions between the monomers. Polar interactions are shown as dashed lines. B. Dimer observed for the afRio1/ATP/Mn^2+^ complex as determined by analysis of crystal structure PDB code 1ZP9, with monomer in magenta oriented similar to monomer A in Fig. 3. C. Dimer observed for the afRio1/adenosine (Ade) complex as determined by analysis of crystal structure PDB code 1ZTF, with monomer in yellow oriented similar to monomer A of the afRio1/toyocamycin complex depicted in Fig. 2.

**Figure 7:**
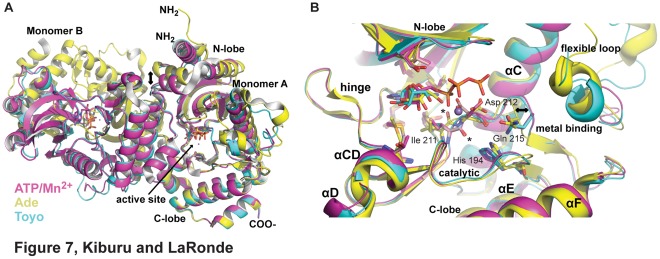
Structural comparison of afRio1 dimers. A. Overlay of the dimers for the adenosine (yellow), toyocamycin (cyan) and ATP/Mn^2+^ (magenta) complexes. The C-lobe (residues 217 to 257) of monomer A were aligned for the superposition. Figure shows the differences in positioning of monomer B, as well as occlusion of the active site by monomer B in the ATP and toyocamycin complexes. In addition, the N-terminal helices of monomer A in the adenosine complexes are shifted (double arrow, 8 to 9 Å) relative to the other complexes. B. Conformational switch observed by the active site of all three complexes (colored as in A with monomer A aligned). A peptide flip is observed that repositions the carbonyl oxygen of Ile 211 (indicated by *; ∼140° rotation, 3.8 Å shift) as a result of steric clash with the cyano moiety of toyocamycin, and interactions of Asp 212 with metal ion in the ATP complex. The metal binding loops in the ATP and toyocamycin complexes are consequently shifted (double arrows, 1.6 to 2.0 Å). This results in reorganization of active site residues such as Gln 215 and His 194.

The PDBe PISA server [36] was used to analyze the buried surface area calculated between monomers in the crystal structures. According to the PISA analysis, the protein has a low probability of forming larger assemblies in solution based on the identified interfaces. However, our data clearly indicate that higher order oligomers do form in all samples except when autophosphorylated. Therefore, we describe the interfaces observed in the crystal packing as possible interaction interfaces in solution. In the structure of the toyocamycin complex, the largest interface (836.5 Å^2^ per molecule) is observed between the two molecules of the asymmetric unit (Fig. 6A). The ATP/Mn^2+^ and ADP/Mn^2+^ bound crystal structures each have four molecules per asymmetric unit, but employ the same packing interfaces observed in the toyocamycin complex, with two copies of a similar dimer per asymmetric unit (Fig. 6B). The largest interface for both the ATP (766.6 Å^2^) and ADP (768.4 Å^2^) bound structures is the one similar to the largest interface in the toyocamycin complex. This interface positions the helices of the C-terminal lobe of one molecule (monomer A) in contact with both the N- and C-lobes at the opening of the active site of the second molecule (monomer B), almost completely occluding that active site (Fig. 3, 6A and B). The N-lobe helix 2 of monomer B and the C-lobe helix, αF of monomer A interact via hydrogen bonds between B:Glu 23 and B:Glu 22 and A:Arg 237, B:Glu 22 and A:Lys 241, A:Lys 25 and A:Tyr 242. In addition, B:Glu 29 interacts with A:Lys 168 (Fig. 6A). The C-terminal lobe of monomer B interacts with the C-terminal lobe of monomer A via conserved B:Tyr 200, which is in hydrophobic contact with A:Leu 252, A:Lys 253, A:Glu 249 and A:Phe 248 from the terminal α helix of monomer A. In addition, there is a hydrogen bond between B:Glu 162 and A:Arg 233. Glu 162 also participates in a water-mediated hydrogen bond with the 3′ OH of the toyocamycin ribose moiety. This interface is not observed in the crystal structure of afRio1 bound to ADE (Fig. 6C). The largest interface observed in the ADE bound structure (1023.3 Å^2^) instead positions the N-lobe of one monomer (monomer A) between the N- and C-lobes of a symmetry related monomer (monomer B), on one side of the active site, close to the hinge (Fig. 6C). The interface is extensive and positions the first three N-terminal helices of monomer A in contact with monomer B. This interface does not result in occlusion of the active site, and in fact, a second symmetry related molecule positions its flexible linker of one monomer (monomer A), which contains the autophosphorylation site (Ser 108), at the entrance to the active site of monomer B. In summary, different, mutually exclusive, interaction interfaces are observed in the crystal structures of afRio1/ADE complex and the afRio1/toyocamycin complex, but the same packing is observed for afRio1 when toyocamycin, ATP/Mn^2+^, or ADP/Mn^2+^ is bound (Fig. 7A). This suggests that Rio1 may exist in at least two distinct dimeric/oligomeric states, and that the toyocamycin bound afRio1 favors the conformation observed in the ATP bound afRio1. To further support the conformational similarity between ATP and toyocamycin complexes, a root mean square deviation (RMSD) plot of afRio1 bound to ATP and ADE calculated using the afRio1/toyocamycin complex as the reference demonstrates that afRio1/ATP shows less deviation from afRio1/toyocamycin compared to the afRio1/ADE (Fig. S6A). In addition, an RMSD plot of residues within 8 Å from the ligand’s center shows less deviation between the afRio1/ATP and toyocamycin structures (Fig. S6B).

The involvement of Tyr 200 in the interaction interfaces is notable, since Tyr 200 is either Phe or Tyr in all RIO kinases, and is part of the catalytic loop containing the catalytic Asp and Asn residues. In the interfaces identified in the crystal structures of the afRio1 complexes with ATP and toyocamycin, Tyr200 interacts with several residues via hydrophobic contact (Fig. 6A). Since the interfaces observed in the crystal lattice may not be the actual interface in solution, we tested whether Tyr 200 plays a role in dimerization of afRio1 when toyocamycin or ATP is bound. Tyr 200 was mutated to Asp (Y200D) in order to break the predicted hydrophobic interactions in the interface, and determined its oligomeric state by analytical ultracentrifugation in the presence and absence of bound ligand. Sedimentation equilibrium data for the unliganded Y200D mutant fit to an average molecular weight of 70.8±1.1 kDa assuming a single-species model and fit best to a monomer-tetramer model (Table 3, Fig. S4 G-H), indicating that oligomerization is not significantly affected by the mutation in the unliganded form. However, when toyocamycin or ATP/Mg^2+^ is added, the average molecular weight drops to 48.2±1.1 kDa and 46.8±1.1 kDa respectively, with best fit to a monomer-trimer model in both cases indicating that mutation of Tyr 200 compromises the interface in these complexes. The average molecular weights of toyocamycin and ATP/Mg^2+^ Y200D complexes are lower compared to the wild-type complexes in both cases (Table 3). This data demonstrates that the mutation of Tyr 200 compromises oligomerization in the presence of toyocamycin or ATP/Mn^2+^, but has no effect on that of the ADE complex. This supports the idea that these complexes utilize different interfaces for oligomerization, and is consistent with the use of the observed crystallographic interfaces.

### How afRio1 Tetramers Form

In all afRio1 structures, the dimer formed by the interfaces described above would result in open oligomers, in that the interactions are heterologous, formed by two separate surfaces on the molecules. Although this type of interaction is uncommon, it has been observed for other protein kinases, for example Epidermal Growth Factor Receptor (EGFR), which forms an asymmetric dimer prior to activation [37]. This asymmetry means that a second interface is available for higher order oligomer formation for both monomers. Therefore, tetramers observed in AUC experiments could be formed from interaction between dimers. Tetramers predicted based on analysis of crystal contacts for toyocamycin, ATP and adenosine bound forms are shown in supplementary figure S7. Analysis of the adenosine complex revealed that interaction between dimers utilizing the largest interaction interface would bury an additional 1055 Å^2^ of surface area, and present the autophosphorylation site to the active site. For the toyocamycin and ATP complexes, an additional 881 and 819 Å^2^ is buried respectively. Therefore, it is possible that dimer formation nucleates higher order oligomers, and anything that stabilizes the dimer would promote formation of the tetramer. In this model, trimers could be formed by addition of a monomer to a dimer, and would be observed when the proportion of monomer is increased, as seen for autophosphorylated protein in the presence of ATP/Mg^2+^ and toyocamycin.

### AfRio1 Changes Oligomeric Forms via a Conformational Switch

Since toyocamycin is an adenosine analog, and more closely resembles adenosine than ATP, it is surprising that it selects the ATP-bound conformation, and binds more tightly than ATP/Mg^2+^. Therefore, we hypothesized that there must be structural conformational changes conserved between the ATP and toyocamycin bound structures that are absent in the ADE bound structure and influence the choice of interaction interface. Upon closer inspection of the active sites of the structures with adenosine, toyocamycin and ATP bound, we observed a difference in positioning of the loop containing the metal binding residue, Asp 212 (metal-binding loop; Fig. 7B). Specifically, in the adenosine bound structure, the backbone carbonyl oxygen bond of Ile 211 is pointing into the ATP binding pocket, while in the ATP and toyocamycin bound structures, there is a peptide flip that positions the Ile 211 carbonyl oxygen bond in the opposite direction, resulting in a 3.8 Å shift in position of the carbonyl oxygen. A simulated annealing Fo-Fc omit map for the afRio1-toyocamycin complex, calculated after omitting residues 211–212 revealed density for the peptide bond supporting the peptide flip that is observed between the adenosine and toyocamycin complexes (Fig. S8). The change produces a shift in the metal-binding loop containing residues Ile 211 to Gln 215, such that the Cα of Gln 215, a residue that is invariant in all RIO kinases, is shifted by 2.1 Å. The result is a hydrogen bond between the Gln 215 side chain and the Asp 212 carbonyl oxygen in the toyocamycin and ATP structures, and a shift of the flexible loop of afRio1 away from the active site (Fig. 7B). An inspection of the overall alignment of the monomers for the various complexes also reveals a shift in the positioning of the N-lobe relative to the C-lobe in the adenosine complex, resulting in a more closed cleft between them when ATP or toyocamycin is bound (Fig. 7A).

## Discussion

Our results show a tight binding compound, toyocamcin that has high affinity for both *A.fulgidus* and human Rio1 and confers similar conformational changes as those observed in the afRio1/Mn^2+^ complex. Whereas toyocamycin inhibits the maturation of 40 S subunit during ribosome biogenesis [19]–[22], Rio1 is required for the maturation of this small subunit. We therefore present the first identified target of toyocamycin during this process.

In characterizing this system we observe an apparent mixed inhibition that may result from by the formation of oligomers that are less catalytically active. This is supported by both structural analysis and solution-based studies (sedimentation equilibrium and size exclusion) that show the presence and sampling of multiple oligomeric states depending on the ligand bound (toyocamycin or ATP) as well as the phosphorylation state of Rio1. Structural analysis of interacting interfaces in crystal structures of toyocamycin and ATP bound complexes reveal occlusion of the active site in participating monomers and hence the approach of substrate. In effect, both toyocamycin and ATP stabilize an oligomeric form of the protein in which the active site of only one monomer in any oligomer is accessible to the substrate supporting inhibition by oligomerization. Substrate inhibition by high concentrations of ATP could be a biological regulatory mechanism similar to that observed in various enzymes, including kinases such as phosphofructokinase, that employ substrate inhibition as a regulatory feedback mechanism [38]–[41].

Though we can explain inhibition by association of oligomers, it is not clear that we can definitively define the inhibition model due to the complexities of our system. Detailed steady state inhibition experiments with a specific-Rio1 substrate would aid in better explaining the inhibition mechanism. What is conclusive from our data is that afRio1 oligomerization is promoted by ligand-binding, and thus oligomerization may play a role in substrate inhibition and regulation of enzyme activity. This has previously been observed in other enzymatic systems, such as HIV integrase, which is inhibited by a shift in its oligomeric equilibrium from an active form to an inactive tetramer that is promoted by ligand binding [42].

The crystal packing interfaces of all the complexes were analyzed and show similar packing for the toyocamycin and ATP complex, but a distinct set of packing interfaces for the ADE complex, despite toyocamycin’s resemblance to adenosine, suggesting possible quaternary structure for the oligomers. A conformational switch mediated by a single peptide backbone flip at Ile 211 was identified when both the toyocamycin and ATP/Mg^2+^ bound forms were compared to the adenosine complex. This conformational change causes a significant shift of the metal-binding loop. As a result, the perturbation of structure caused by the peptide flip is disseminated to the positioning of the N-lobe and C-lobe relative to each other, and thereby the choice of crystal packing interaction interface is affected. If the crystal packing interface reflects the interfaces used in solution, the peptide flip can be regarded as a switch between two choices of higher order oligomeric states. Regulation of the protein might be enacted by changes in the number of protein molecules in which the switch is flipped on or off through the type and concentration of the ligand. This discovery may indicate that the enzymatic activity of Rio1 is regulated by its oligomeric state. Since the interfaces have not been extensively biochemically validated, we cannot be certain that they are the ones utilized in solution. However, that there exist distinct interfaces is supported by the mutation of Tyr 200 that affects oligomerization for toyocamycin and ATP complexes, but not the ADE complex.

The data presented in this analysis suggest that Rio1 kinase shows some of the characteristics of a morpheein [43], [44]. The morpheein model of allostery was first described for human porphobilinogen synthase, and is characterized by more than one distinct monomer conformations in the dissociated state, and multiple distinct quaternary assemblies from these as a result [45], [46].

In an effort to assign a role of Rio1 autophosphorylation, we analyzed the effect of phosphorylating Rio1 and discovered that autophosphorylation promotes monomerization. Steady state kinetics experiments indicate that this form is more active than the unphosphorylated afRio1 based on the maximum catalytic velocity (V_max_). Therefore afRio1 activity may be regulated by its oligomerization properties and autophosphorylation promotes the more active free form, the monomer. Human Rio1 could function in a similar manner as it is evident that autophosphorylated hRio1 promotes monomerization to some extent. In fact, Angermayr and colleagues have shown that yeast Rio1 kinase can by phosphorylated by Casein Kinase II and this phosphorylation results in activation and degradation of the protein in a cell cycle phase dependent manner [47]. Recent reports also indicate that hRio1 kinase activity is necessary for association with precursors of 40 S and is required for recycling the endonuclease hNob1 and the dimethyltransfase hDim2 [48]. This could mean that the hRio1 oligomeric state could be regulated both by autophosphorylation and phosphorylation by other kinases. In addition, Rio1 has been shown to participate in the formation of protein complexes in the cell [49], and regulation of its oligomeric state may also influence interaction with other proteins. Taken together, these data would assign a role for autophosphorylation in modulating the activity of Rio1 kinase, both in enzymatic activity and in protein-protein interactions.

Finally we present the first crystal structure of toyocamycin bound to its first identified ribosome processing target, Rio1. The tight binding of toyocamycin to the afRio1 ATP binding pocket provides a tool that can be used in future experiments to identify Rio1 substrates by various methods that utilize radiolabeled ATP analogs to capture kinase substrates [50]–[53]. A more recent unbiased validation assay that couples both functional proteomics and chemical genetics for identifying kinase substrate using inhibitors can also be employed using our system [54].

## Supporting Information

Figure S1 Thermal shift curves for four compounds (Toyocamycin, ATP, Sangivamycin and 7-methylinosine ) that were screened for binding to afRio1. Each curve represents one of the three replicate reactions. Toyocamycin (red) shows the largest shift in melting temperature.
(TIF)

Figure S2 Alignment of afRio1-toyocamycin complex’s active site indicating an almost perfect superposition of the active site of the two molecules found in the asymmetric unit.
(TIF)

Figure S3 Overlay of afRio1/toyocamycin (green) and afRio1/ATP (purple) binding sites shows that both ligands bind to the same site. Residues in the binding site are labeled.
(TIF)

Figure S4 Sedimentation equilibrium data collected at three different speeds; 18,000 rpm (blue dots), 22,000 rpm (green dots), and 26,000 rpm (red dots). The fitted data was analyzed at all three speeds and at the same concentration (0.25 µg/µl). The residuals for each fit are provided in the top panel and show random distribution in most. A. AfRio1 with adenosine bound. B. AfRio1 with ATP bound. C. AfRio1 with toyocamycin bound. D. AfRio1 with ADP bound. E. Phosphorylated afRio1 with Toyocamycin bound. F. Phosphorylated afRio1 with ATP bound. G. The afRio1 Y200D mutant with no ligand bound. H. The afRio1 Y200D mutant with toyocamycin bound.
(TIF)

Figure S5 Residuals for sedimentation equilibrium data collected at three different speeds; 18,000 rpm (blue dots), 22,000 rpm (green dots), and 26,000 rpm (red dots). The fitted data was analyzed at all three speeds and at the same concentration (0.25 µg). A-C. AfRio1 with Toyocamycin bound fitted to: A. monomer-dimer, B. dimer, C. monomer-trimer. D. AfRio1 fitted to monomer-dimer. E. AfRio1 fitted to dimer. F. AfRio1 with ATP bound fitted to monomer. G. AfRio1 with ADP bound fitted to monomer. H. Phosphorylated afRio1 with ATP bound fitted to monomer. I. Phosphorylated afRio1 with ATP bound fitted to monomer-dimer. J. Phosphorylated afRio1 with toyocamycin bound fitted to monomer. K. Phosphorylated afRio1 with toyocamycin bound fitted to monomer-dimer. L. AfRio1 Y200D mutant fitted to monomer-dimer. M. AfRio1 Y200D fitted to monomer-trimer. N. AfRio1 Y200D mutant with ATP bound fitted to a monomer. O. AfRio1 Y200D mutant with ATP bound fitted to monomer-dimer. P. AfRio1 Y200D mutant with toyocamycin bound fitted to a monomer. Q. AfRio1 Y200D mutant fitted to monomer-dimer.
(TIF)

Figure S6 Plots of the root mean square deviations (RMSD) for afRio1 complexes. All calculations were carried out using the toyocamycin-bound structure as the reference. A. RMSD comparisons over all residues of afRio1 bound to ATP and adenosine (ADE). Larger deviations are observed with the adenosine structure. B. RMSD comparison of residues within 8 Å radius from the ligand’s center. The plots demonstrate that the afRio1-ATP structure show smaller deviations from the toyocamycin bound structure.
(TIF)

Figure S7 Tetramers predicted from crystal packing interactions for the afRio1/toyocamycin, afRio1/ATP and afRio1/ADE tetramers. A. AfRio1/ATP tetramer with ATP bound to each monomer. B. AfRio1/toyocamycin tetramer with toyocamycin bound to each monomer (similar to the afRio1/ATP tetramer). **C.** AfRio1/adenosine tetramer with adenosine bound to each monomer (different from A. and B.). Tetramers were generated by displaying symmetry related molecules with the largest and second largest buried surface area between them.
(TIF)

Figure S8 A simulated annealing Fo-Fc omit map calculated after omitting residues 211–212 (contoured at 2.5σ) reveals electron density for the Ile 211-Asp 212 peptide bond in the afRio1-toyocamycin complex (cyan). The toyocamycin (cyan) and ATP (magenta) show the backbone carbonyl oxygen bond of Ile 211 pointing away from the ATP-binding pocket. The associated peptide bond is flipped in the afRio1-adenosine complex (yellow), which results in the carbonyl oxygen bond pointing into the binding pocket.
(TIF)

Table S1 Sedimentation Equilibrium (Variance)^1/2^ for different oligomeric state models absorbance Plots.
(DOC)
