# Supplementary figures and images for: Interaction of Rio1 Kinase with Toyocamycin Reveals a Conformational Switch That Controls Oligomeric State and Catalytic Activity
Source: PLoS One. 2012 May 22;7(5):e37371. doi: 10.1371/journal.pone.0037371 (PMC3358306; doi:10.1371/journal.pone.0037371)

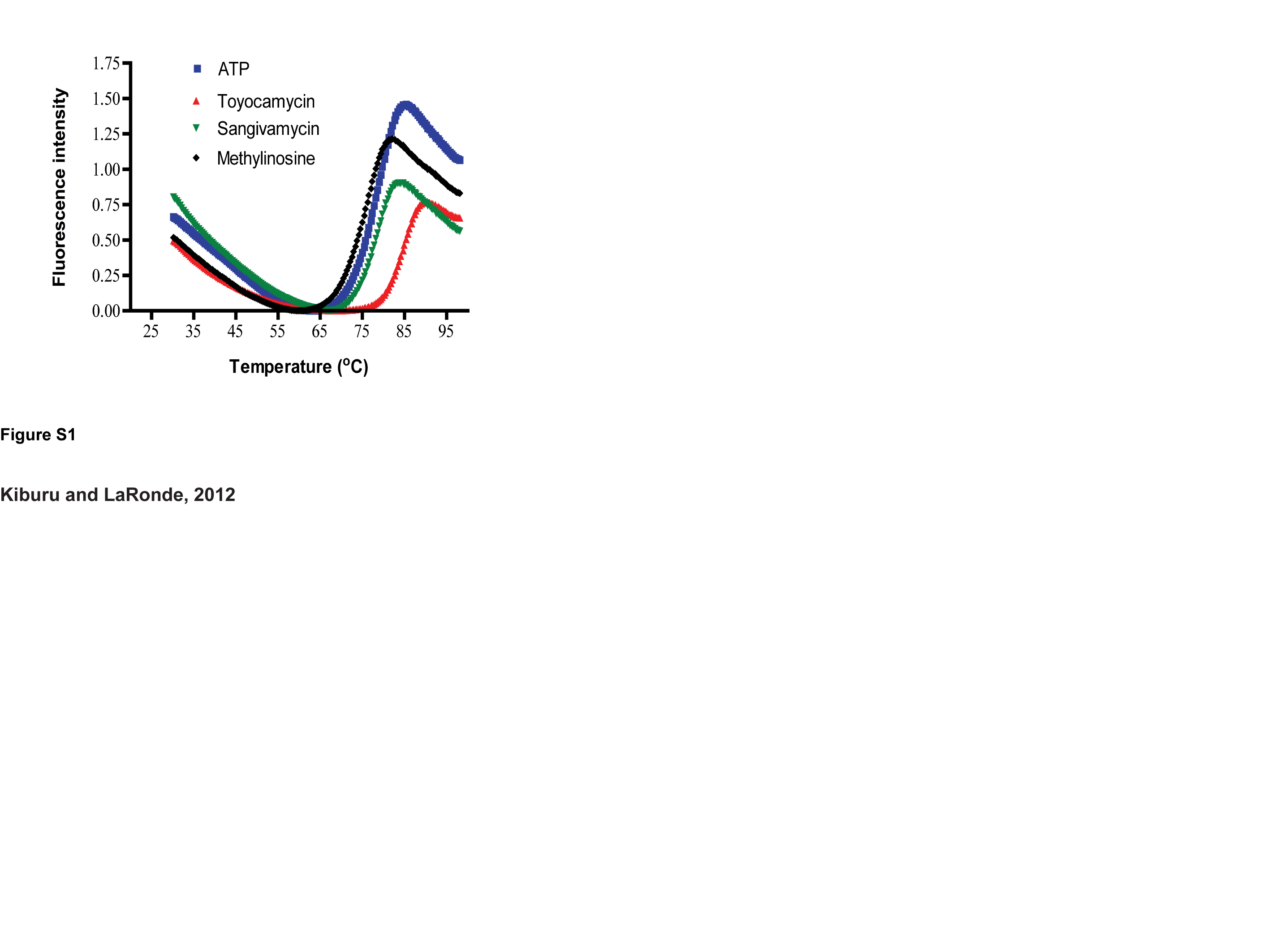

Supplement: Figure S1 — Thermal shift curves for four compounds (Toyocamycin, ATP, Sangivamycin and 7-methylinosine ) that were screened for binding to afRio1. Each curve represents one of the three replicate reactions. Toyocamycin (red) shows the largest shift in melting temperature. (TIF) [file pone.0037371.s001.tif]

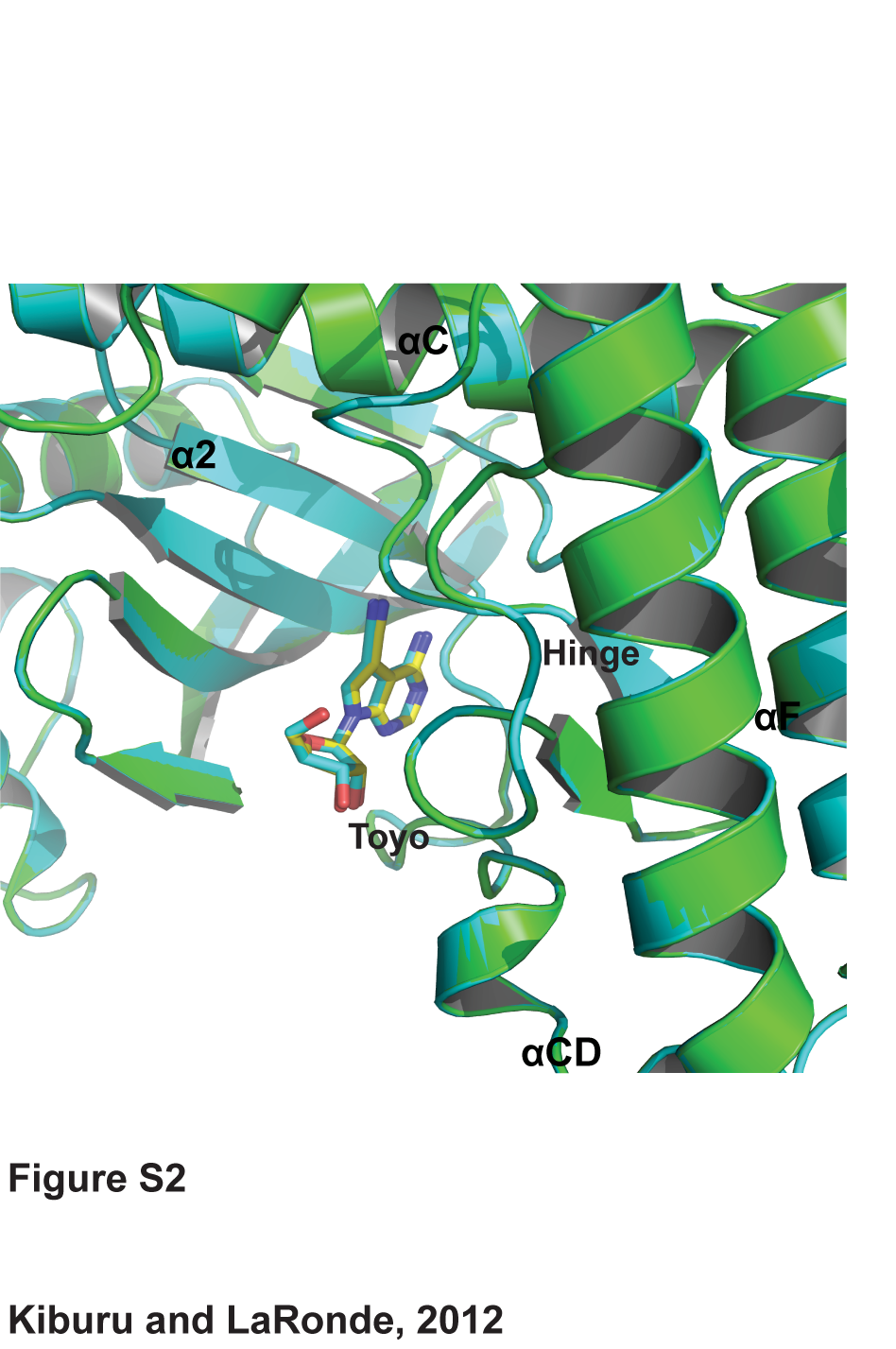

Supplement: Figure S2 — Alignment of afRio1-toyocamycin complex’s active site indicating an almost perfect superposition of the active site of the two molecules found in the asymmetric unit. (TIF) [file pone.0037371.s002.tif]

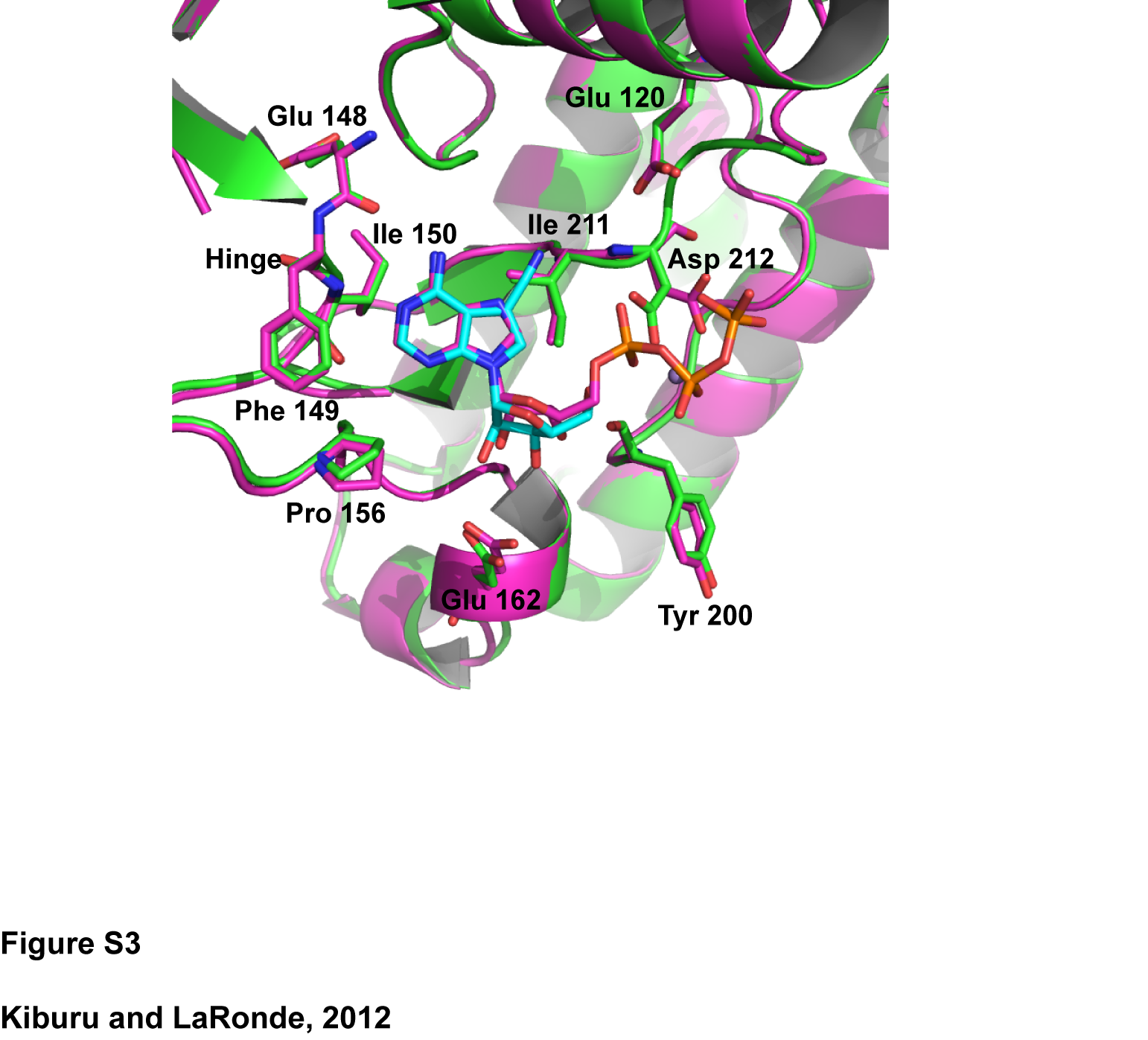

Supplement: Figure S3 — Overlay of afRio1/toyocamycin (green) and afRio1/ATP (purple) binding sites shows that both ligands bind to the same site. Residues in the binding site are labeled. (TIF) [file pone.0037371.s003.tif]

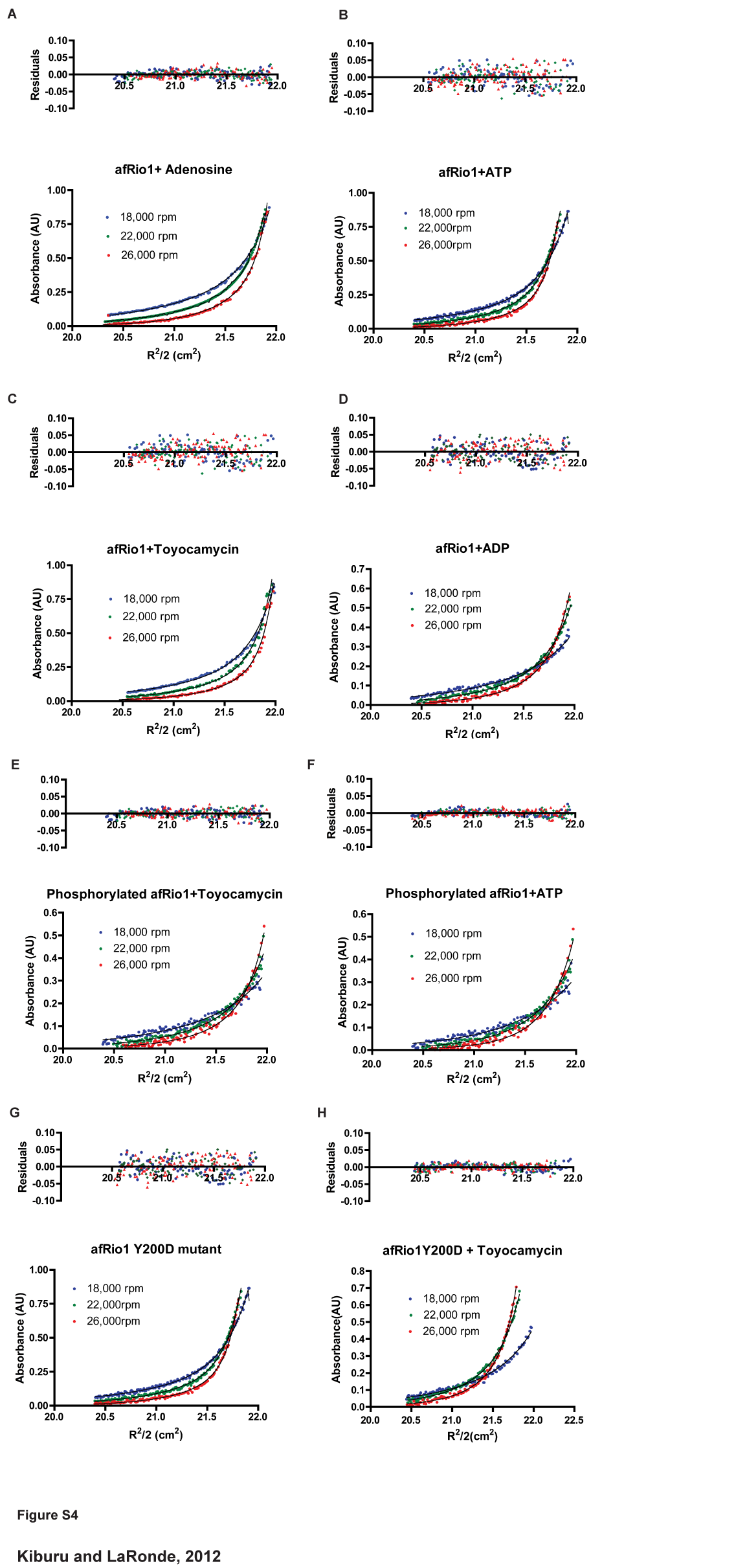

Supplement: Figure S4 — Sedimentation equilibrium data collected at three different speeds; 18,000 rpm (blue dots), 22,000 rpm (green dots), and 26,000 rpm (red dots). The fitted data was analyzed at all three speeds and at the same concentration (0.25 µg/µl). The residuals for each fit are provided in the top panel and show random distribution in most. A. AfRio1 with adenosine bound. B. AfRio1 with ATP bound. C. AfRio1 with toyocamycin bound. D. AfRio1 with ADP bound. E. Phosphorylated afRio1 with Toyocamycin bound. F. Phosphorylated afRio1 with ATP bound. G. The afRio1 Y200D mutant with no ligand bound. H. The afRio1 Y200D mutant with toyocamycin bound. (TIF) [file pone.0037371.s004.tif]

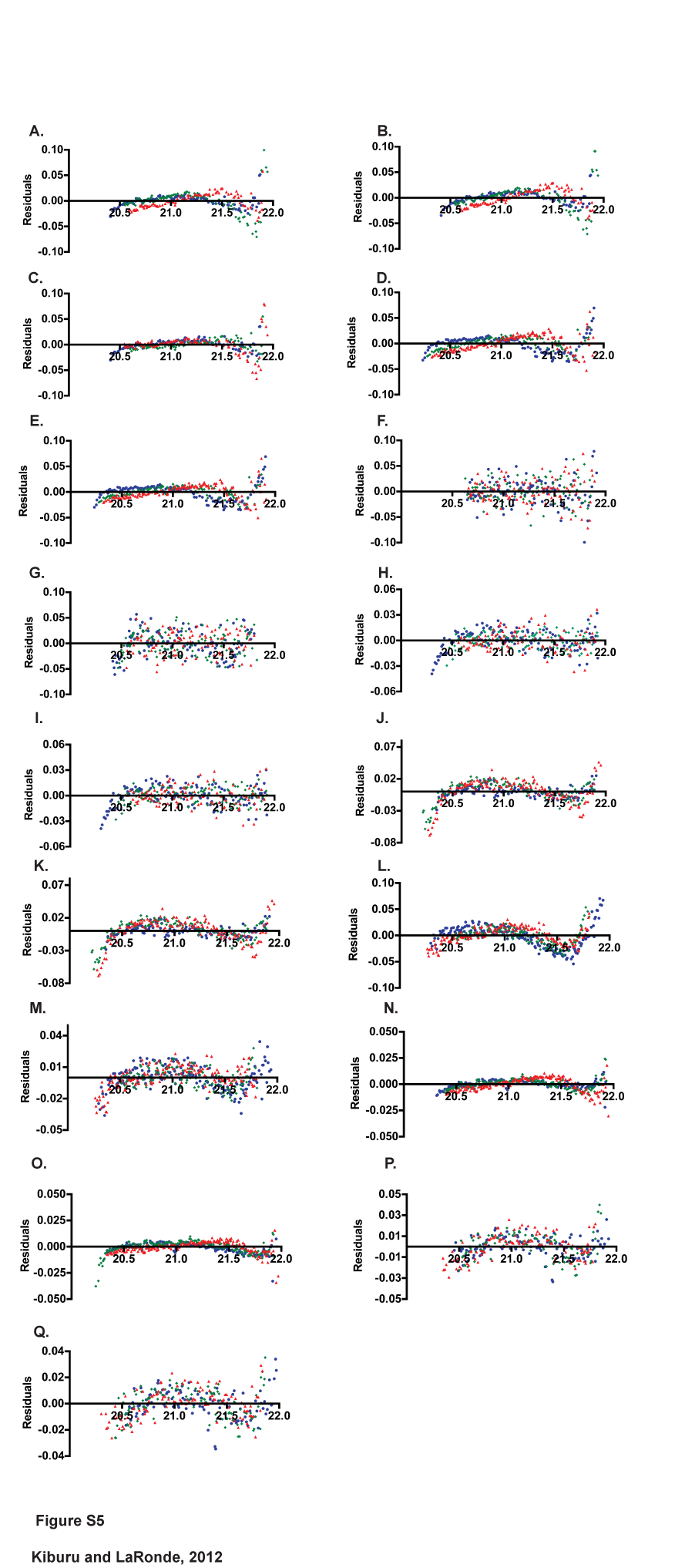

Supplement: Figure S5 — Residuals for sedimentation equilibrium data collected at three different speeds; 18,000 rpm (blue dots), 22,000 rpm (green dots), and 26,000 rpm (red dots). The fitted data was analyzed at all three speeds and at the same concentration (0.25 µg). A-C. AfRio1 with Toyocamycin bound fitted to: A. monomer-dimer, B. dimer, C. monomer-trimer. D. AfRio1 fitted to monomer-dimer. E. AfRio1 fitted to dimer. F. AfRio1 with ATP bound fitted to monomer. G. AfRio1 with ADP bound fitted to monomer. H. Phosphorylated afRio1 with ATP bound fitted to monomer. I. Phosphorylated afRio1 with ATP bound fitted to monomer-dimer. J. Phosphorylated afRio1 with toyocamycin bound fitted to monomer. K. Phosphorylated afRio1 with toyocamycin bound fitted to monomer-dimer. L. AfRio1 Y200D mutant fitted to monomer-dimer. M. AfRio1 Y200D fitted to monomer-trimer. N. AfRio1 Y200D mutant with ATP bound fitted to a monomer. O. AfRio1 Y200D mutant with ATP bound fitted to monomer-dimer. P. AfRio1 Y200D mutant with toyocamycin bound fitted to a monomer. Q. AfRio1 Y200D mutant fitted to monomer-dimer. (TIF) [file pone.0037371.s005.tif]

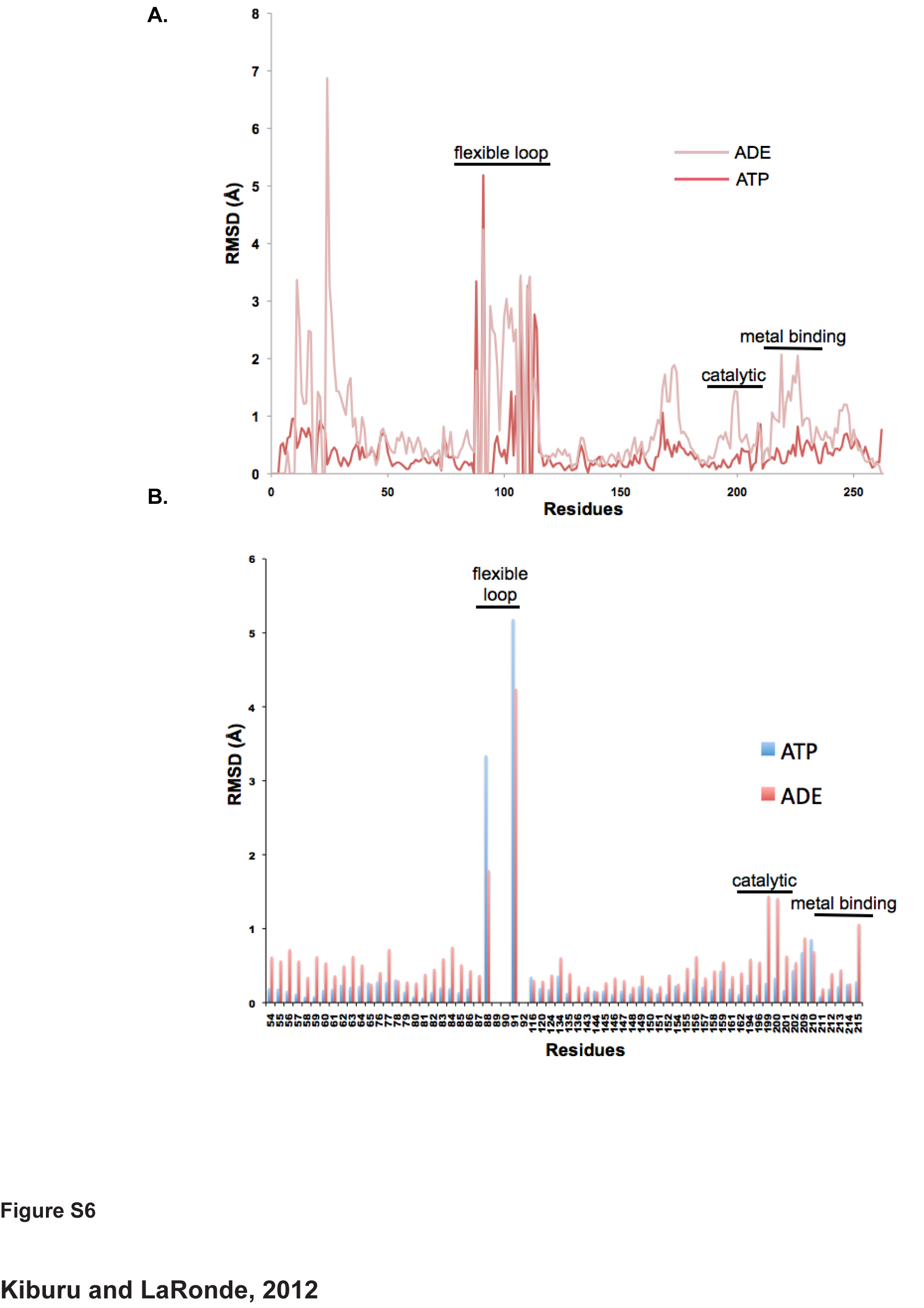

Supplement: Figure S6 — Plots of the root mean square deviations (RMSD) for afRio1 complexes. All calculations were carried out using the toyocamycin-bound structure as the reference. A. RMSD comparisons over all residues of afRio1 bound to ATP and adenosine (ADE). Larger deviations are observed with the adenosine structure. B. RMSD comparison of residues within 8 Å radius from the ligand’s center. The plots demonstrate that the afRio1-ATP structure show smaller deviations from the toyocamycin bound structure. (TIF) [file pone.0037371.s006.tif]

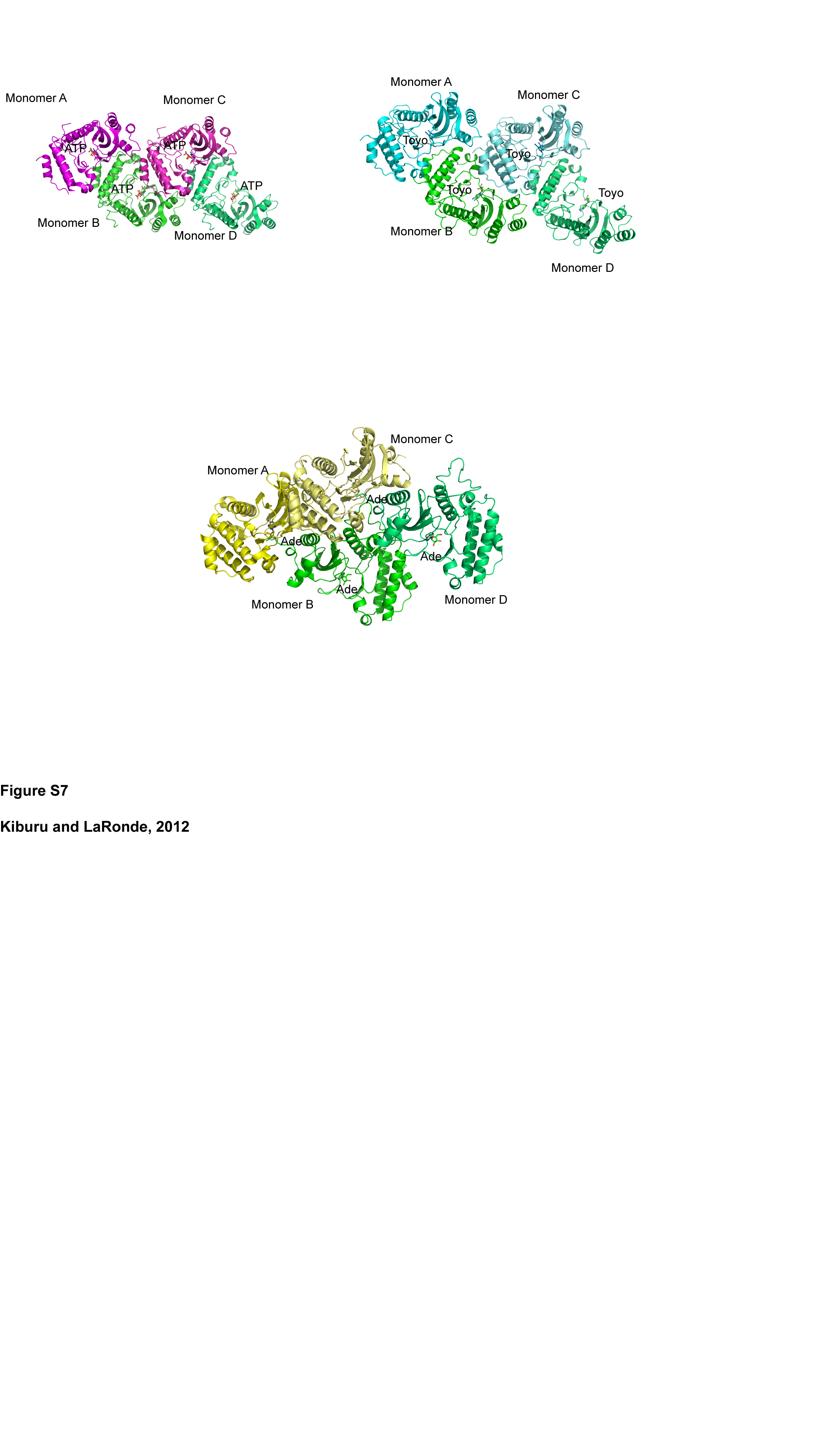

Supplement: Figure S7 — Tetramers predicted from crystal packing interactions for the afRio1/toyocamycin, afRio1/ATP and afRio1/ADE tetramers. A. AfRio1/ATP tetramer with ATP bound to each monomer. B. AfRio1/toyocamycin tetramer with toyocamycin bound to each monomer (similar to the afRio1/ATP tetramer). C. AfRio1/adenosine tetramer with adenosine bound to each monomer (different from A. and B.). Tetramers were generated by displaying symmetry related molecules with the largest and second largest buried surface area between them. (TIF) [file pone.0037371.s007.tif]

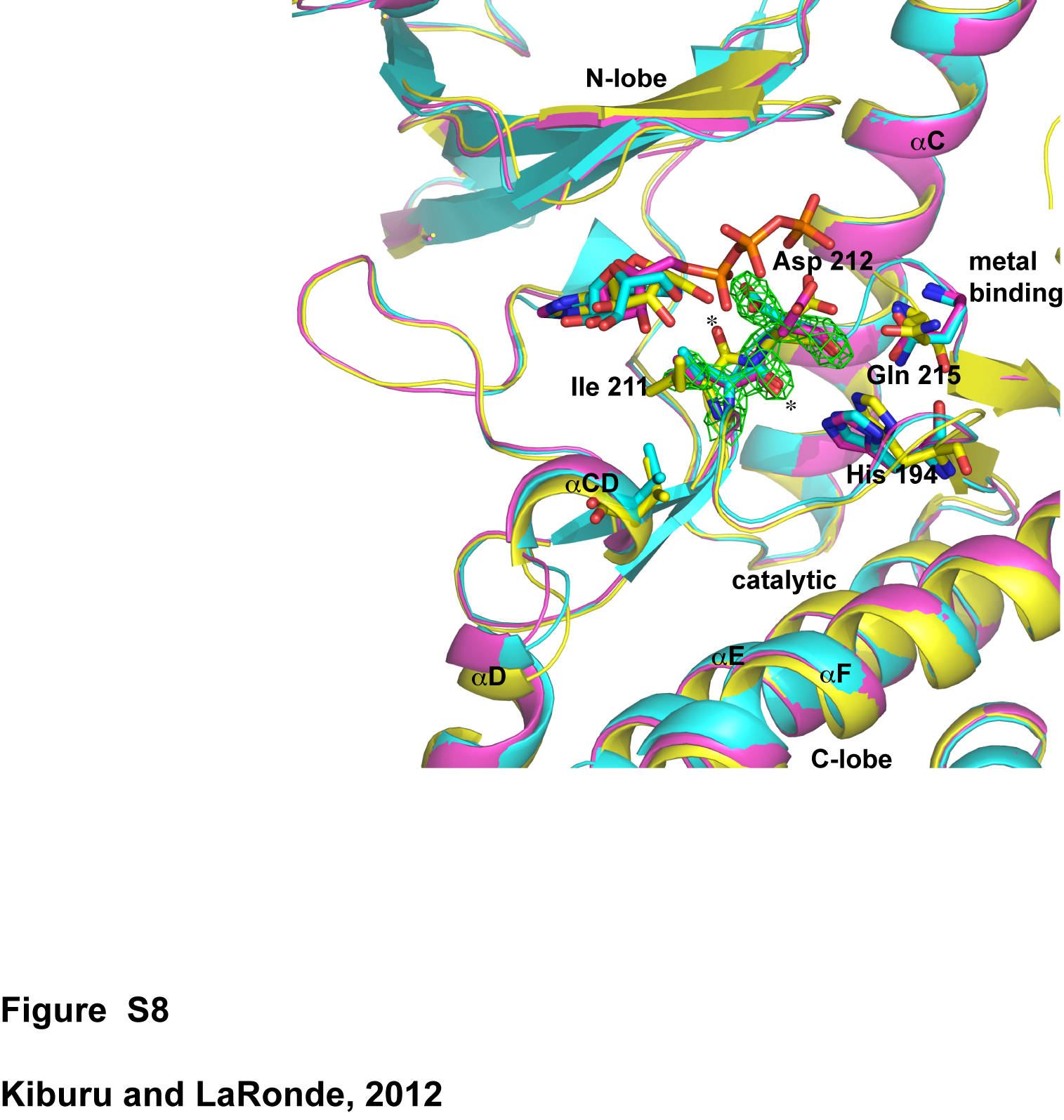

Supplement: Figure S8 — A simulated annealing Fo-Fc omit map calculated after omitting residues 211–212 (contoured at 2.5σ) reveals electron density for the Ile 211-Asp 212 peptide bond in the afRio1-toyocamycin complex (cyan). The toyocamycin (cyan) and ATP (magenta) show the backbone carbonyl oxygen bond of Ile 211 pointing away from the ATP-binding pocket. The associated peptide bond is flipped in the afRio1-adenosine complex (yellow), which results in the carbonyl oxygen bond pointing into the binding pocket. (TIF) [file pone.0037371.s008.tif]
