# Supplementary material for: Interaction of Rio1 Kinase with Toyocamycin Reveals a Conformational Switch That Controls Oligomeric State and Catalytic Activity
Source: PLoS One. 2012 May 22;7(5):e37371. doi: 10.1371/journal.pone.0037371 (PMC3358306; doi:10.1371/journal.pone.0037371)
Supplement: Table S1 — Sedimentation Equilibrium (Variance)1/2 for different oligomeric state models absorbance Plots. (DOC) [file pone.0037371.s009.doc]

**Table S1**

| **Sample** | **Monomer** | **Monomer-Dimer** | **Dimer** | **Monomer-Trimer** | **Monomer-Tetramer** |
| --- | --- | --- | --- | --- | --- |
| AfRio1 | N/A | 1.4 x 10^-2^ | 1.6 x 10^-2^ |  | **8.0 x 10^-3^** |
| Afrio1 + toyocamycin | N/A | 2.9 x 10^-2^ | 2.8 x 10^-2^ | 2.4 x 10^-2^ | **1.8 x 10^-2^** |
| AfRio1+ ATP | 3.0 x 10^-2^ | **2.8 x 10^-2^** | N/A | N/A | N/A |
| AfRio1 + ADP | 2.8 x 10^-2^ | **2.4 x 10^-2^** | N/A | N/A | N/A |
| Phosphorylated afRio1+ATP | 1.1 x 10^-2^ | 1.1 x 10^-2^ | N/A | **8.8 x 10^-3^** | N/A |
| Phosphorylated afRio1+Toyocamycin | 1.6 x 10^-2^ | 1.5 x 10^-2^ | N/A | **1.3 x 10^-2^** | N/A |
| AfRio1_Y200D | N/A | 3.34 x 10^-2^ | N/A | 1.1 x 10^-2^ | **9.2 x 10^-3^** |
| AfRio1_Y200D+ATP | 1.1 x 10^-2^ | 1.0 x 10^-2^ | N/A | **9.4 x 10^-3^** | N/A |
| AfRio1_Y200D +Toyocamycin | 1.1 x 10^-2^ | 1.0 x 10^-2^ | N/A | **9.3 x 10^-3^** | N/A |

“N/A” indicates model used resulted in non-convergence. All fits were performed using the program WinNonlin. In bold represents the best model fit based on the lowest (variance)^1/2^ that correlates to residuals.

**Kiburu and LaRonde, 2012**
